# Expectations in culturally unfamiliar music: influences of proximal and distal cues and timbral characteristics

**DOI:** 10.3389/fpsyg.2013.00789

**Published:** 2013-11-07

**Authors:** Catherine J. Stevens, Julien Tardieu, Peter Dunbar-Hall, Catherine T. Best, Barbara Tillmann

**Affiliations:** ^1^The MARCS Institute, University of Western SydneySydney, NSW, Australia; ^2^OCTOGONE - PETRA, Université de Toulouse Le MirailToulouse, France; ^3^Conservatorium of Music, The University of SydneySydney, NSW, Australia; ^4^Haskins LaboratoriesNew Haven, CT, USA; ^5^Lyon Neuroscience Research Center, CNRS-UMR 5292, INSERM U1028, Université de LyonLyon, France

**Keywords:** expectations, timbre, tuning, gamelan, cross cultural

## Abstract

Listeners' musical perception is influenced by cues that can be stored in short-term memory (e.g., within the same musical piece) or long-term memory (e.g., based on one's own musical culture). The present study tested how these cues (referred to as, respectively, proximal and distal cues) influence the perception of music from an unfamiliar culture. Western listeners who were naïve to Gamelan music judged completeness and coherence for newly constructed melodies in the Balinese gamelan tradition. In these melodies, we manipulated the final tone with three possibilities: the original gong tone, an in-scale tone replacement or an out-of-scale tone replacement. We also manipulated the musical timbre employed in Gamelan pieces. We hypothesized that novice listeners are sensitive to out-of-scale changes, but not in-scale changes, and that this might be influenced by the more unfamiliar timbre created by Gamelan “sister” instruments whose harmonics beat with the harmonics of the other instrument, creating a timbrally “shimmering” sound. The results showed: (1) out-of-scale endings were judged less complete than original gong and in-scale endings; (2) for melodies played with “sister” instruments, in-scale endings were judged as less complete than original endings. Furthermore, melodies using the original scale tones were judged more coherent than melodies containing few or multiple tone replacements; melodies played on single instruments were judged more coherent than the same melodies played on sister instruments. Additionally, there is some indication of within-session statistical learning, with expectations for the initially-novel materials developing during the course of the experiment. The data suggest the influence of both distal cues (e.g., previously unfamiliar timbres) and proximal cues (within the same sequence and over the experimental session) on the perception of melodies from other cultural systems based on unfamiliar tunings and scale systems.

## Introduction

Lifetime exposure to a particular musical environment enables listeners to acquire expectations for rhythmic or metric patterns (Hannon and Trehub, 2005a), tonal and harmonic structure (Schmuckler, 1989; Bigand and Pineau, 1997), and melodic structure (Carlsen, 1981; Cuddy and Lunney, 1995). But what happens when a listener encounters novel musical scales and timbres that fall outside those familiar, culturally-tuned musical structures? One possibility is that culturally unfamiliar modalities are perceived through the framework of the cultural system with which one is already familiar (Curtis and Bharucha, 2009). That is, when listening to music from an unfamiliar modal system, it may well be that listeners' own cultural expectations (that is expectations based on knowledge about musical features that are acquired over a relatively long time span; referred to hereafter as “distal cues”) are imposed on that new musical system. A second possibility is that listeners attune readily to the sensory cues and statistical regularities (referred to hereafter as “proximal cues”) of a new musical context (e.g., Bharucha, 1987; Loui et al., 2010; Creel, 2011).

Here, we investigated both the distal cues and proximal cues hypotheses as they may apply to the perception of melodies composed in an unfamiliar musical tradition in which instruments are tuned to unfamiliar scales and make use of unfamiliar timbres. Listeners familiar with a Western tonal tradition were exposed in the laboratory to melodies composed in the *selisir* mode of the Balinese *pelog* scale and played on Balinese gamelan instruments, and were asked to make judgments about the completeness and coherence of those melodies. The scale, intervals, timbre, and tuning of the *selisir* mode of the Balinese *pelog* differ from the diatonic scale of the Western system. This material thus provides an ecological medium for the investigation of experimental hypotheses developed most often for Western tonal (and timbral) material, but applied here to culturally unfamiliar material.

### Characteristics of balinese gamelan

Characteristics of Balinese Gamelan are related to the melodic structures and the scale system used, as well as to the instruments with their particular tuning and timbral quality. The melodic structure of gamelan music differs from that of Western tonal music. In keeping with the Balinese worldview, the music is organized into repeating cycles and epicycles that come together at the sound of the large gong (Kessler et al., 1984, p. 135). Balinese music is regenerative, with the music returning to the same point; the last note of a melody is the first note when it recurs, and this “moment of renewal” is signified by a stroke of the large gong. The tuned instruments provide the melodic content of the music (Tenzer, 1998; Gold, 2005). As major melodic sections in a piece end on the *gong tone* (literally, the tone played by the gong instrument in the gamelan ensemble), the gong tone thus shares some similarities with the tonic tone of the Western diatonic scale (Kessler et al., 1984, p. 140; Gold, 2005).

Regarding the scale system, there are two sets of guidelines: *pelog* and *slendro*. *Pelog* is a seven-note system consisting of a series of unequal intervals. The large (L) and small (S) intervals between successive tones have the pattern SSLSSSL; this pattern of intervals is the complement of the Western diatonic scale—LLSLLLS (Kessler et al., 1984, p. 140). Rather than all seven unequally spaced tones of the *pelog* being used in a single composition, groups of five or four are isolated to form modes. A piece is usually composed in a particular mode and the tones in the mode are labeled with one of the Balinese solfa names *ding, dong, deng, dung*, and *dang*. There are eight modes that can be derived from the *pelog* system, such as for example *selisir* (Tenzer, 2000). While the resulting patterns are similar to permutations of the SLSSL pattern of the pentatonic scale, the sizes of the intervals in the *pelog* modes differ from interval sizes in the Western pentatonic modes (Kessler et al., 1984, p. 140). As well as *pelog* modes differing according to which five of the seven tones are included in each mode, they also differ regarding which of the five tones takes the role of the *gong tone*, which has a referential function in this tone set. Stimulus materials in the present experiment were created using the *selisir* mode of the *pelog* system.

**Figure 1:**
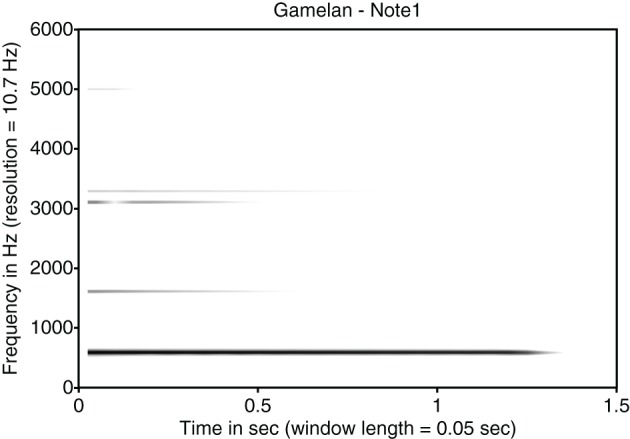
**Spectrogram of a note from the Gangsa instrument**. The spectrogram shows a lowest frequency partial at 599 Hz and three inharmonic partials at ~1621, 3114, and 3296 Hz.

Balinese music is played by a group of musicians in an ensemble of instruments called a gamelan. In a bronze gamelan, instruments consist of resonant metal bars suspended above resonators, and gongs. Gamelans are tuned to a particular scale for that set of instruments—supposedly, no two gamelans are tuned identically, thus each gamelan ensemble has a unique “voice quality” (tuning). Tenzer (1998) writes that “each set of instruments has its own characteristic sound and tonal personality” (p. 31). Such flexibility generates the variety of tunings in actual practice in Bali. The instruments are struck with wooden, cloth covered, or rubber mallets. When struck, the instruments produce complex overtones quite different from the harmonic series of frequencies of Western string and wind instruments. To illustrate the overtones of a note played on the *Gangsa* instrument of the Balinese gamelan, a spectrogram of a note from Gangsa is shown in Figure 1. The gamelan spectrogram shows a lowest frequency partial at 599 Hz added to three other inharmonic partials at ~1621, 3114, and 3296 Hz. These three partials are very weak in energy compared to the lowest frequency partial. In addition to the inharmonic nature of individual notes, each of the different bronze key instruments of Balinese gamelan occurs in instrument pairs with a particular tuning relation within the pair. Tenzer (1998) notes that most gamelans beat at between 5 and 8 Hz (see Appendix 1). The beat frequency is due to a frequency difference between each note of sister instruments of around 7 Hz. It is the beats produced by paired or “sister” instruments that give rise to the shimmering quality of the gamelan ensemble. Melodies played by these paired instruments are referred to as “sister melodies” hereafter.

### Enculturation effects in music perception

Enculturation refers to the perceptual attunement that results from exposure to a particular environment (e.g., Gibson and Gibson, 1955; Gibson, 1969; Pfordresher and Brown, 2009; Stevens et al., 2013). A number of studies have demonstrated that culture-specific experience affects the perception of musical structure. Enculturation to the Western tonal scale has been demonstrated even for young children (Lynch et al., 1991; Trainor and Trehub, 1992). Lynch et al. (1991) proposed that enculturation involves the development of a tonal scheme that acts as a perceptual lens. When presented with an unfamiliar scale, a mismatch occurs between unfamiliar scale intervals and the acquired scheme, however, the listener tends to perceive the unfamiliar scale as a (perhaps odd) exemplar of their culturally acquired scheme. Thus, the listener may fail to detect a critical change in a note for a melody composed in the culturally-unfamiliar scale. Culturally-specific perceptual reorganization for musical tuning appears to begin to affect perception sometime between 6 and 12 months of age (Lynch and Eilers, 1992). Similar enculturation development has also been observed in Western listeners for metrical structures in music; it is not yet evident at 6 months of age, but can be observed at 12 months of age (Hannon and Trehub, 2005a,b).

One way that expectations for music are acquired is through a process of statistical learning (e.g., Krumhansl, 2000; Krumhansl et al., 2000; Tillmann et al., 2000; Eerola, 2004). In everyday life, such learning can occur through repeated exposure to music of a particular culture—the *distal* context—compatible with the preceding description of enculturation. In the laboratory setting, statistical learning for new material can be shown even over very short time scales, either across experimental trials or within-trial sensory priming, i.e., in more *proximal* musical contexts.

Expectations based on listeners' long-term musical knowledge acquired through mere exposure in everyday life—the distal context—have been studied with the priming paradigm investigating speed of processing and with rating judgments. The priming paradigm showed that in Western listeners (e.g., American) listening to chords in the Western musical tradition that are harmonically related to a prime are processed more quickly and with fewer errors than chords that are less related to the prime (Bharucha, 1987; Tillmann, 2005). Similarly, rating judgments have shown greater sensitivity to the tonal hierarchy in North Indian music in Indian listeners as compared to American university students (Castellano et al., 1984; see also Dowling, 1984). Results of regression analysis by Castellano et al. (1984) revealed that ratings given by Indian listeners were based, in part, on listeners' long-term musical knowledge about the musical genre, that is, musical enculturation.

The distribution of tones in the immediately prior context in an experimental setting or the proximal context also influences listeners' perception. In an unfamiliar cultural framework, participants receiving exposure to the material within an experimental trial draw on sensory memory, with judgments based on what fits or does not fit the preceding context. For example, presenting Balinese music to Western and Balinese participants, Kessler et al. (1984) demonstrated that both groups were sensitive to a hierarchy of tonal function, scale membership, and frequency of occurrence of tones in the context melody. Variability in responding, however, increased as familiarity with the musical culture decreased, indicating some interaction between proximal and distal influences in perception. Similarly, results of Castellano et al. (1984) further revealed that the American students became sensitive to features related to the tone occurrence in the preceding context; they lack the influence of expectations based on distal cues (no enculturation to Indian music), but they can nonetheless make some use of the influence of proximal cues within the short-term context of the experimental materials.

Research using artificial material with new tonal scales and structures has confirmed that exposure to musical material within an experiment leads to rapid on-line learning: using artificial scales and a probe-tone task, Creel and Newport (2002) showed that when participants rated how likely it was that the probe tone had been contained in the preceding melody, they rated more highly those tones that had occurred more frequently, and were also sensitive to the tone in the final position. Creel (2011) demonstrated that exposure to a particular context shifted listeners' preferences toward probes matching the context with which they had been familiarized. She concluded that listeners rapidly form specific musical memories without explicit instruction, which are then activated during music listening. Loui et al. (2010) used finite state grammars and the Bohlen-Pierce scale to generate melodies to which musician listeners were passively exposed. Thirty minutes of exposure led to recognition, generalization and sensitivity to event frequencies and grammatical structures and increased preference for repeated melodies in the new system. Similarly, Tillmann and Poulin-Charronnat (2010), combining implicit learning with the priming paradigm, have shown that after exposure to tone sequences based on a finite state grammar, non-musician listeners use this newly acquired knowledge to develop expectations for future tones in a grammatical sequence, which then influence speed of processing.

### The present study

Building on these various findings, the perception as well as the on-line learning of proximal cues is tested here in the context of culturally unfamiliar material—with unfamiliar distal cues of scale and timbre—and musically untrained (i.e., non-musician) listeners. In a first phase of the experiment, Australian (Western musical culture) participants rated completeness of Balinese Gamelan melodies where the final note was the conventional scale tone of a *selisir* mode (the gong tone) or an alternative note to the gong tone, selected from within the particular scale or outside the scale. Within Balinese Gamelan conventions, melodies ending on the gong tone should sound maximally complete. We investigated the tendency for listeners unfamiliar with the Gamelan scales to rate as more complete the melodies that end on the gong tone compared with those that end on another in-scale tone (also from the *selisir mode)* or an out-scale tone (unrelated and not belonging to the *selisir* mode). To measure the effects of manipulating these melodic expectations, listeners rated how complete the melody sounded (e.g., Boltz, 1989; Bigand and Pineau, 1997 for use of these judgments for Western tonal material). If the proximal context and sensory cues influence judged completeness of the melody, then ratings of melodies with no-change endings (i.e., the original gong tone ending) should be similar to ratings of melodies with in-scale changes, which, in turn, should both be greater than ratings of melodies with out-of-scale changes.

A second way to measure sensitivity to culturally unfamiliar music is to disrupt the statistical conventions and associated expectations by replacing the gong tone as it occurs during the unfolding of a melody with an out of scale tone and have listeners rate the coherence of the melody. Coherence refers to the entire melody in contrast with completeness, which refers to the relationship between the final tone and the preceding melodic context. In judging coherence, listeners rate how coherent the melody sounds relative to other melodies they know. Two levels of disruption were made with partial or total replacement of the gong tones. Partial replacement of the gong tone as it occurs during the melody is a relatively subtle change with few instances of the gong tone replaced. By contrast, total replacement refers to all instances of the gong tone in the melody being replaced. This is a less subtle change. Based on the assumption that the notes of the mode cohere, then total replacement of the gong tone should elicit relatively lower ratings of coherence compared with partial replacement. In a second phase of the experiment, listeners judged coherence of these melodies. If there is sensitivity, even in novice listeners, to the tone set of a melody, then melodies without replacement will be rated more highly than those with partial or total gong tone replacements. Contrasting with Creel and Newport (2002), the scale will be ecological rather than artificial and played on instruments with novel timbre and tuning.

Previous studies have manipulated the cultural background of listeners as an independent variable. This is effective in investigations of bimusicalism (e.g., Wong et al., 2009, 2011), but also introduces variability in that groups familiar with a particular World music such as Gamelan or Indian music, because of globalization (Huron, 2008), are also enculturated to Western tonal music. As an alternative and given the lack of monomusical Balinese listeners, we manipulated musical material to investigate effects of distal and proximal contexts, within the experimental setting, on Western listeners' judgments of completeness and coherence on melodies composed in a Balinese scale and played on Balinese instruments. In this way, we are able to gauge long-term influences of Western enculturation and more immediate effects of within-session exposure to experimental stimuli on musical completeness and coherence of unfamiliar music played with an unfamiliar timbre. Non-musicians were recruited, as they have implicitly acquired some basic knowledge of Western tonal music (Bigand and Poulin-Charronnat, 2006; Hannon and Trainor, 2007; Ettlinger et al., 2011). For example, adult non-musicians are sensitive to the tonal hierarchy in Western tonal music (Krumhansl, 2004), to key and harmony (Trainor and Trehub, 1994), and to classes of emotional response often associated with major or minor modes and fast or slow tempo (Balkwill et al., 2004; Filipic et al., 2010). As Wong et al. (2011) commented, musical exposure is often passive listening rather than performing. Thus, examining listeners without formal training allows investigation of effects of passive exposure without active use (see also Bigand and Poulin-Charronnat, 2006).

By sampling from the non-musician population rather than musicians who may have had more exposure to diverse and less familiar timbres, and less familiar scales that use different intervals, the unfamiliar nature of the Gamelan timbre is optimized with the tones and melodic relations differing maximally from notes of the Western tonal scale played on an instrument with familiar timbre. Tuned percussion instruments, such as the metallo-percussive instruments of a brass Gamelan ensemble, differ from (more Western) wind and string instruments in that they do not naturally generate harmonically tuned overtones (Fletcher and Rossing, 1991). Such inharmonic sounds, which produce a variety of pitch sensations (i.e., more varied than sounds with F0 and harmonics, Zwicker and Fastl, 1999), will be less familiar to non-musicians than musicians where the latter will likely have had more exposure to diverse timbres. This expectation of enhanced unfamiliarity of Gamelan instrument tonal quality is consistent with research on pitch perception, which has revealed that non-musicians, compared with musicians, are particularly influenced in pitch judgments by harmonics or overtones rather than by F0 (Seither-Preisler et al., 2007; McLachlan et al., 2013). More simply, pitch perception is better for more familiar timbres, even in non-musicians. To investigate the effect of the characteristic shimmer or beating of Gamelan sister instruments on judgments of completion and coherence in the present study, we included melodies played on a single instrument and the same melodies played on “sister” instruments. One prediction is that the more dissimilar the timbral quality of the melody is from Western timbre (i.e., with the sister instrument timbre), the lower the ratings of completion and coherence by non-musicians should be.

### Design and hypotheses

There were two parts to the experiment. The independent variables were timbre material (a between-subjects factor: single melody, sister melody) and in Part 1, ending tone type (no change-original gong tone, in-scale replacement, out of scale replacement), and in Part 2, tone set (original, partial replacement, total replacement of the gong). The dependent variables were ratings of melody completeness (Part 1) and coherence (Part 2). It was hypothesized that: (1) if proximal cues influence judged completeness, then ratings of melodies with no-change endings should be similar to ratings of melodies with in-scale changes which, in turn, should both be greater than ratings to melodies with out-of-scale changes; (2) if listeners are sensitive to the tone set of a melody then higher ratings of coherence should be given to melodies without gong tone replacements followed by those with partial and then total replacement changes; and (3) ratings of completeness and coherence should differ between single instrument melodies and sister instrument melodies. Specifically, increased dissimilarity with familiar Western timbre (and thus the distal context) should lead to lower ratings of completion and coherence.

## Method

### Participants

Thirty students from the University of Western Sydney participated in this experiment: 15 were assigned to the single-melody condition and 15 to the sister-melody condition. They reported having had no prior musical training on any musical instrument, except two participants (one in each group) who indicated 2 and 6 months, respectively. In a post-questionnaire, participants were asked whether they had ever listened to gamelan music and, more specifically, to Balinese gamelan music. All participants indicated “no” to both questions.

### Stimuli

Ten novel *pelog*-scale melodies were composed according to Balinese gamelan composition protocols by an expert in this musical tradition (author PDH), designed to cover the five possible modes and ending on the relevant gong tone, with two melodies for each gong tone. The 10 original melodies depicted using Cipher notation are shown in Appendix 2. Auditory examples of the stimuli can be found here (http://katestevens.weebly.com/stimuli.html). In the original melodies, the final tone was thus the gong tone and was the same as the first tone of the melody (i.e., respecting the Balinese system). For the out-of-scale endings, the gong tone was replaced by a tone outside the scale of the melody. This out-of-scale tone thus had not occurred in the melody before and represented a deviant based not only on the scale structure, but also on sensory features (i.e., a new event). This was made possible through the use of instruments from a *gamelan samara dana*, a type of Balinese ensemble tuned to the full seven-tone pelog scale. This type of gamelan differs from most other types of Balinese gamelans, which are tuned to only the four of five notes of a mode of pelog. The Gangsa instrument was used because all of the tones could be played on the one instrument controlling changes in harmonics and spectral content that would have occurred if seven notes had been played on two different instruments.

For the in-scale endings, the gong tone was replaced by a different tone belonging to the scale of the melody (see Appendix 2). The melodies were composed and the replacement tones were chosen in such a way that the in-scale tone did not introduce sensory violations with the preceding melodic context in comparison to the gong tone. For that aim, the following characteristics were controlled and compared between the gong tone (original ending) and the in-scale ending. The frequency of occurrence (weighted by duration) of the final tone (i.e., the gong or the in-scale tone) in the preceding melodic context did not differ for the gong-condition and the in-scale condition (9.4 ± 4.55 for gong vs. 11.85 ± 3.35 for in-scale, *p* = 0.16). The intervals created by the last tone and the penultimate tone were not equal between the two conditions, but did not differ significantly in size between the gong condition and the in-scale condition (2.7 ± 2.41 vs. 4.6 ± 2.01, *p* = 0.13). These intervals did not differ in melodic contour, except for two melodies where the final tone was repeated (i.e., one melody ending on the gong and one on the in-scale tone). With the hypothesis that the penultimate tone might trigger expectations for the final tone based on chunks (i.e., tone pairs) occurring in the melodic context, we also calculated the frequency of occurrence of the last two tones, which were considered as a “bigram” (the final tone and the penultimate tone), in the melodic context. The frequency of this tone chaining did not differ for the gong condition and the in-scale condition (1.7 ± 1.83 vs. 1.4 ± 2.27, *p* = 0.74).

Melodies for the coherence judgments: The original melodies were composed in different characters using various motifs. Descriptions of some of the melodic characteristics and motifs can be found in Appendix 2. The number of gong tones replaced by an out of scale tone was on average 2.8 ± 1.32 (ranging from 1 to 5) for the “partial replacement” condition and 7.4 ± 3.57 (ranging from 2 to 12; replacing all instances) for the “total replacement” condition. These replacements differed significantly, *p* = 0.0003 (with paired *t*-test over the 10 melodies). For the partial replacements, the replaced tones could occur either at the beginning (in 4 melodies), in the middle (3 melodies), or at the end (3 melodies).

### Equipment and stimulus constraints

**Figure 2:**
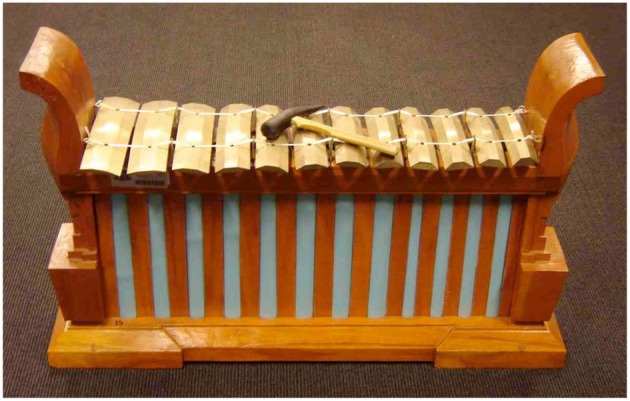
**Gangsa instrument**. Photo: Peter Dunbar-Hall.

Tokens of tones from the *selisir* mode of *pelog* were studio-recorded in mono using ProTools with a 24 bit, 96K sampling rate and an AKG C414(B) cardioid microphone and 40 Hz filter. The instrument was *Gangsa* (Figure 2) and was played using a rubber mallet by author PDH who is highly experienced in gamelan performance and musicianship. A number of takes were recorded for each of the nine notes—notes 1 to 7 including two notes from a lower octave (L) but from the same instrument. Each of the notes was recorded for 5 different note durations: 4 beats (i.e., 2400 ms; semibreve), 3 beats (dotted minim), 2 beats (minim) 1.5 beats (dotted crotchet), 1 beat (i.e., 600 ms; crotchet), and 0.5 beat (quaver). Dotted quavers were excised from performance of a segment rather than played as a single note. Each gamelan melody was played in its entirety to give a sense of the whole melody and provide a reference point for the ideal sound and flow when note items were sequenced to form complete melodies in addition to the single tone recording, as described next. The stimulus melodies for perceptual tests were formed from single note concatenation via MIDI, as explained below.

#### Creating the melodies

From the recording session, the best tokens of each note were selected. Based on the global sound quality, the quality of the attack, and the homogeneity between all the tokens, 19 tokens were selected to create the melodies. The 19 tokens were normalized and loaded into the built-in sampler (EXS24) of LogicPro. A maximum of 3 different tokens per note was used, and chosen randomly during the playing of a melody. This was done to achieve a more natural-sounding performance using more than one sample per note (this method is commonly used in sample-based virtual instruments). A damping envelope was applied to the end of the sound to control the duration of the sample (100 ms linear damp). The 50 melodies for the single-melody condition (i.e., the 10 original melodies and their different versions, that is, with in-scale and out-of-scale endings as well as with partial and total replacement changes) were written in Logic as MIDI tracks and played using the sampler.

A new set of sound files was created for the sister-melody condition by mixing the melodies played with the original instrument with the same melodies played with a simulation of the sister instrument. The simulation consisted in a pitch-shifting of each note of the original instrument using the pitch-shift algorithm with preservation of the spectral envelope available in the software Audiosculpt^1^. In other words, the simulated sister instrument had the same spectral structure as the original instrument, but all the frequencies were shifted. There is no resampling in Audiosculpt, rather pitch is shifted with conservation of the duration using fast Fourier transform (FFT) and inverse FFT. Two sound files here (http://katestevens.weebly.com/stimuli.html) demonstrate that the manipulation does not alter the timbre of the sound. In order to get the necessary amount of pitch shifting for each note, the sister instrument was recorded with the performer playing the same nine notes as for the original instrument. The recording used the AKG C414(B) microphone, MOTU 896 HD firewire audio interface, a MacBook and the software MAX/MSP. Each note of the sister instrument was analyzed in Audiosculpt in order to find the pitch of each note in Hz. The same operation was performed on the original instrument in order to calculate the pitch difference in Hz (see Appendix 1). Then, each note of the original instrument was pitch-shifted to the appropriate value using Audiosculpt, to create a “simulation” of the sister instrument, to ensure that the duration and tonal dynamics of each sister tone were identical to those of the corresponding original tone. This effect was applied to the 19 tokens selected previously. The 50 melodies were then played in LogicPro by both the original and the sister instruments synchronously and exported as audio files.

#### Creating stimulus blocks

Two types of test blocks were created according to the type of modifications made to the melodies: Block 1 (containing the original melodies and the modified versions with their in-scale and out-of-scale endings) and Block 2 (i.e., the original melodies and the modified versions with either partial or total replacement). Each block was 30 melodies long and made using five different random orders. The constraint for the random order was to have a minimum of one different melody between two versions of a given melody. For example, dong(1) could never be followed or preceded by dong(1) in its in-scale version or its out-of-scale version. Each melody was followed by a 3 s silence, a 100 ms 2000 Hz pure tone (a “beep”), and 2 s silence. Participants had 5 s to make their judgment, with the beep signaling the last 2 seconds. Each melody was preceded by 500 ms of white noise and 1 s silence. Each version of the two blocks was then exported in.aiff format to be played during the experiment from a CD. A set of nine practice melodies was created using the same method.

### Procedure

Participants read an information sheet and signed a consent form in accordance with the University of Western Sydney Human Research Ethics Committee approval. The experimenter explained to small groups of participants that the task investigated responses to unfamiliar melodies. In the first part of the experiment, participants rated using a seven-point scale how complete the melody sounded to them (relative to other melodies they know), and in the second part how coherent the melody sounded to them (relative to other melodies they know). The steps along the scale for Completeness ran from (1) Weakly complete through to (7) Very complete, and for Coherence from (1) Weakly coherent to (7) Very coherent. To set a musical and cultural context, a 30 s excerpt of Balinese gamelan music was played from a CD (“Gambang” from the CD Bali: Gamelan Semar Pegulingan: Gamelan Of The Love God) at the beginning of the experiment. In practice trials, included to familiarize participants with the task and procedure, they listened to a melody and then placed a cross on the rating scale at the number that reflects how complete they thought the melody sounded and then in part 2, how coherent the melody sounded. There was a short break between Part 1: judging completeness and Part 2: judging coherence. After the experiment trials, a Background Questionnaire was completed. The experiment took 30 min.

## Results

Participants' completion and coherence judgments were averaged over trials in each condition and were analyzed, respectively, using 3 × 2 ANOVAs, with Material (single melody, sister melodies) as a between-participants factor and either Ending Type (original gong, in-scale replacement, out-of-scale replacement) as the within-participants factor for the completeness judgments, or Tone Set (original, partial replacement, total replacement) as the within-participants factor for the coherence judgments.

### Completion judgments

**Figure 3:**
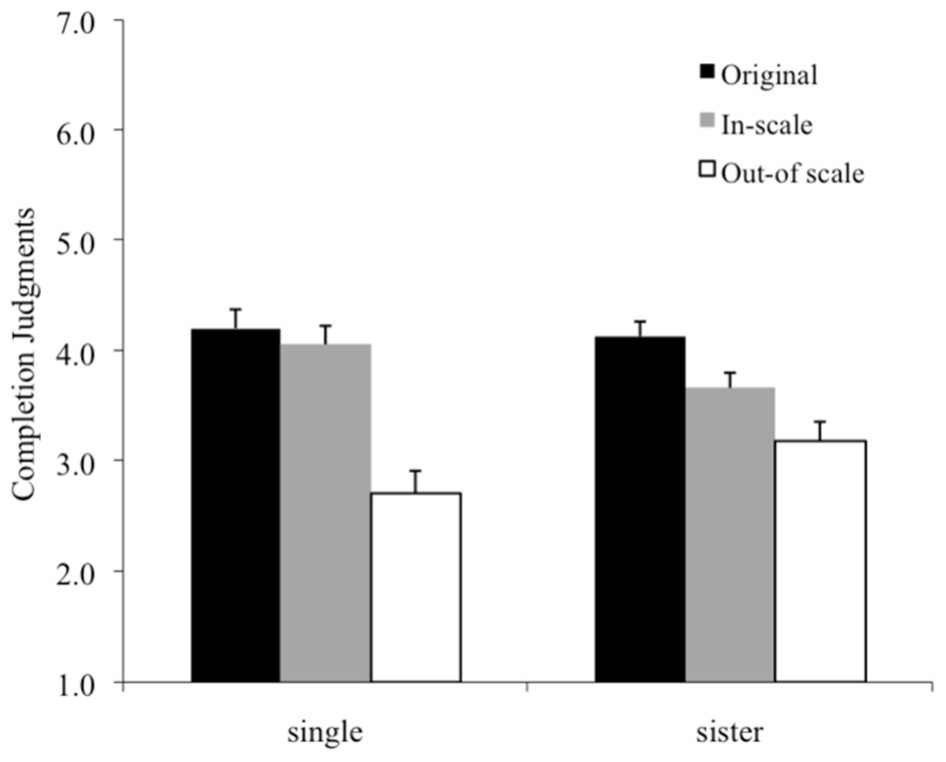
**Mean completion ratings shown as a function of Ending Type and Timbre Material**. The maximum possible rating is 7. Error bars refer to standard errors of the mean.

For completion judgments, the main effect of Ending Type was significant, *F*_(2, 56)_ = 30.79, MSE = 0.39, *p* < 0.001, showing that the out-of-scale endings were judged less complete than the original gong and in-scale endings. The interaction between Ending type and Material was significant, *F*_(2, 56)_ = 3.71, MSE = 0.39, *p* = 0.03 (Figure 3). Melodies were judged as less complete with out-of-scale endings than with the original gong for both single melodies [*F*_(1, 28)_ = 29.83, *p* < 0.001] and sister melodies [*F*_(1, 28)_ = 11.86, *p* = 0.002]. However, the in-scale endings were judged as less complete than the original gong only for sister melodies, [*F*_(1, 28)_ = 9.56, *p* = 0.004], but not for single melodies, *p* = 0.34. The main effect of material was not significant, *F* < 1, *p* = 0.98.

To further investigate whether the participants might have become sensitive to the gamelan scale structure (especially to the difference between the original gong endings and in-scale endings) over the time of the experimental session, completion judgments were separated for the first half and second half of trials in the experimental session, and the differences were evaluated with paired two-sided *t*-tests. For the sister melodies, the ratings between original ending and in-scale endings did not differ in the first half [mean ± SD: 3.93 ± 1.18 vs. 4.06 ± 0.63, *t*_(14)_ = 0.50, *p* = 0.63], but differed in the second half [4.01 ± 0.50 vs. 3.32 ± 0.68, *t*_(14)_ = 3.48, *p* = 0.004]. For the single melodies, however, the ratings between original endings and in-scale endings differed neither in the first half [4.65 ± 0.98 vs. 4.51 ± 0.83, *t*_(14)_ = 0.58, *p* = 0.57], nor in the second half [4.02 ± 0.73 vs. 3.73 ± 0.92, *t*_(14)_ = 1.23, *p* = 0.24]. Thus, the sister melodies but not the single tone melodies yielded some perceptual adaptation during the course of the experiment.

### Coherence judgments

**Figure 4:**
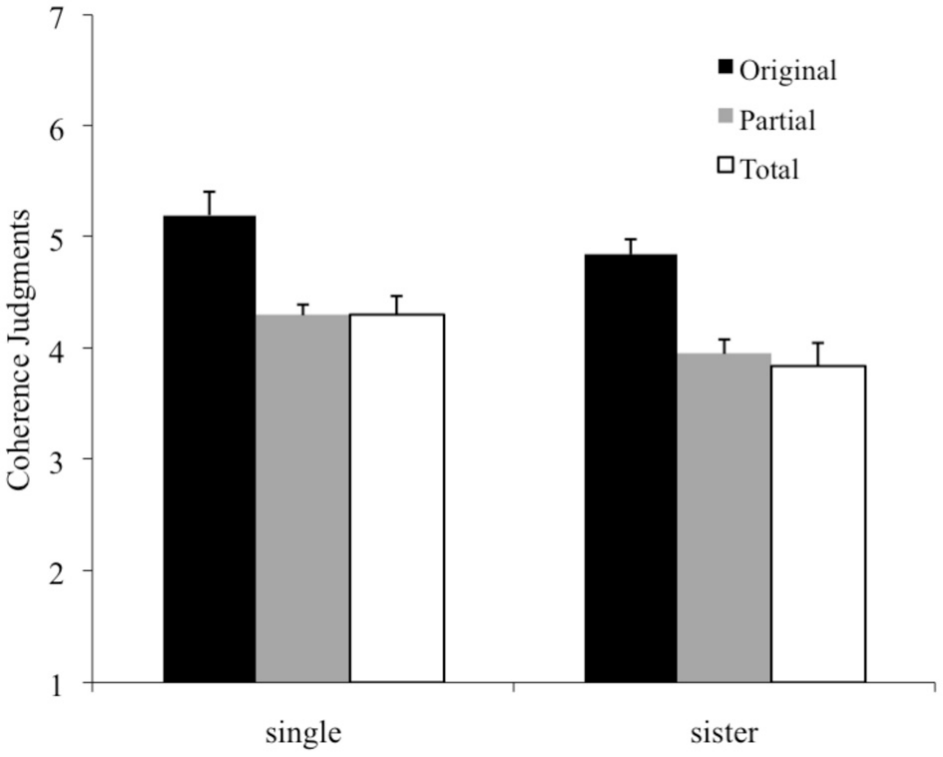
**Mean coherence ratings shown as a function of Tone Set and Timbre Material**. The maximum possible rating is 7. Error bars refer to standard errors of the mean.

For coherence judgments, the main effect of Tone Set was significant, *F*_(2, 56)_ = 26.98, MSE = 0.31, *p* < 0.001, showing that melodies only using the original scale tones were judged as more coherent than melodies containing partial or total replacements. The main effect of Material was also significant, *F*_(1, 28)_ = 6.43, MSE = 0.59, *p* = 0.017, with single melodies being judged more coherent than the sister melodies (Figure 4). Thus, again, the timbral difference between the single and the sister tones reliably influenced performance. The interaction between Tone set and Material was not significant, *F* < 1, *p* = 0.94.

To investigate whether the partial and total replacements might have been judged differently over the course of the experimental session, coherence judgments were separated for the first half and second half of trials, and the difference assessed by paired two-sided *t*-tests. For sister melodies, the ratings for partial replacement indicated higher coherence than ratings for total replacements in the first half (3.69 ± 0.67 for partial vs. 3.23 ± 1.19 for total), even though this difference fell short of significance [*t*_(14)_ = 2.02, *p* = 0.06]; but differences between the two average ratings decreased in the second half [3.69 ± 0.51 for partial, 3.74 ± 0.94 for total, *t*_(14)_ = 0.27, *p* = 0.79]. Similarly for the single melodies, the ratings for partial replacement indicated higher coherence than ratings for total replacements in the first half (partial = 4.47 ± 0.68 vs. total = 3.95 ± 0.96), even though this difference fell also short of significance [*t*_(14)_ = 2.09, *p* = 0.06]; but in the second half, the difference between the two average ratings reversed, but again did not differ significantly [*t*_(14)_ = 1.71, *p* = 0.11, partial = 4.03 ± 0.88, total = 4.43 ± 0.89].

## Discussion

The present experiment investigated effects of immediate memory (or proximal cues) and enculturation (or distal cues) on gamelan-naïve Western listeners' judgments of completion and coherence for melodies composed in the Balinese gamelan tradition. Proximal cues were at play in the finding that out-of-scale endings were judged less complete than the original gong and in-scale endings. Ending type interacted with material such that melodies with out-of-scale endings were judged less complete than original gong tone endings for single and sister melodies, but in-scale endings were judged less complete than original gong tone endings only for sister melodies. The addition of the sister instrument may have increased sensitivity of the Western listeners to the novelty of the gamelan scale structure, as evidenced by the finding that the in-scale endings of sister instrument melodies were judged less complete than original endings in the second half of the completion task trials. Melodies with original scale tones were judged more coherent than melodies with partial or total gong tone replacements. The shimmering timbre of sister instrument tuning influenced judgments of coherence, as well, with sister instrument melodies judged less coherent than corresponding single instrument melodies.

The assignment of significantly greater ratings of completeness for original (gong) melody endings and in-scale endings, as compared to out-of-scale endings, can be explained by proximal cues. The surrounding context enabled listeners to be sensitive to the notes of the mode in which the melody was written and to pick the out-of-scale ending as an unexpected deviant. As Bharucha (1987) noted, immediate prior context can enhance the consonance of a given tone. The present results accord with the theory that listeners rapidly form specific musical memories without explicit instruction (Creel, 2011), and they become sensitive to event frequencies even within an experiment session (Kessler et al., 1984; Loui et al., 2010; Tillmann and Poulin-Charronnat, 2010).

Exposure to the experimental materials enabled abstraction of regularities, such as the role of the gong tone. In construction of the stimulus material, frequencies of occurrence of the gong-tone and the tone used for the in-scale endings were controlled. Such controls enable us to conclude that participants are not simply becoming sensitive to differences in frequencies of occurrence between in-scale and gong-tone endings or differences embedded in less frequent tone transitions or unusual intervals. Rather, participants appear to have picked out some difference in the use of the gong-tone and the in-scale tone within the melodies, and became sensitive to the gong tone as having a more structurally relevant role (beyond frequency or similarity) and making it more appropriate to end the melody. It is plausible that, as Curtis and Bharucha (2009) observed, listeners impose on the expected ending some structural expectations based on their native musical system (i.e., distal cues). Indeed, for the Western tonal musical system, the tonic has been attributed the function of a cognitive reference point that other tones are processed in relation to, providing maximum closure (Krumhansl, 1990). As this feature of a “central tone” has been observed also in other cultural musical systems and has been suggested as a cognitive universal across musical systems (e.g., Dowling and Harwood, 1986; Stevens and Byron, 2009), listeners might adapt this feature also to the new unfamiliar material that they have been exposed to in the present experiment.

In the experiment, the coherence judgments always followed completion judgments so participants had, by that time, heard the material a number of times. The total tone replacements and the more subtle partial tone replacements made to the stimuli in the coherence judgment trials were both sufficient to disrupt the listeners' expectations of the melody. In addition, we observed that the information linked to the instrument timbre also influenced participants' coherence judgments. Coherence was judged to be weaker for the unfamiliar timbre of the sister instrument melodies compared with the single instrument melodies. That is, the timbre that was more unfamiliar to Western listeners (i.e., less enculturation to Gamelan instrument timbre) was associated with lower ratings of coherence.

For completion judgments, the most extreme changes—out-of-scale versus original endings—were easily discerned by listeners independently of the timbral implementation, in that lower judgments of completeness were assigned to melodies ending on the out-of-scale tone than the original endings. However, the finer difference between in-scale and original endings was only detected for the shimmering sister melodies, but not for the single instrument melodies. In contrast to disturbing perception, the unfamiliar timbre seems to help participants to pick out the new regularities over the experimental session. This has been revealed by the additional analyses suggesting within-session perceptual learning. By the second half of the completion judgment trials, participants without musical training and naïve to Balinese gamelan music were judging in-scale endings as less complete than original gong tone endings. One interpretation is that rather than a perceptual or enculturation bias operating and hindering the processing of the new structured material, the sister instrument timbre, which is more ecologically valid for the musical system under investigation, facilitated learning of the scale structure and perception of the original gong endings. That is, the more unfamiliar timbre allowed listeners to be more receptive to the new structured material, with less perceptual bias from cultural knowledge linked to the Western tonal system. A definitive answer along these lines requires repeating the experiment with a sample of trained Western musicians, as well as with trained Balinese gamelan musicians as controls. In addition, the overlapping spectrum of the sister instruments may have enhanced the perceptual judgments. As 7 Hz differences are unresolvable by the auditory nerve, such an effect could only be due to beating or co-modulation within each critical band. Either the overlapping spectra of sister instruments enhanced the extraction of the structurally important gong tone, and/or the unfamiliar sister instrument timbre reduced the perceptual bias, thus giving an impression of perceptual sensitivity to the gong tone and to the violation of completeness when a tone other than the gone tone ended the piece. While separation of the two influences is not possible with the present experiment, demonstration of such influences is a first step in bringing new questions into relief for future investigation.

The results suggest influences from enculturation (e.g., the functional role of a reference tone) and short-term (within experiment) statistical learning in cross-cultural music perception. In addition, the familiarity of instrument *timbre* has been shown to play a role in melody perception and perceptual learning. As an alternative to manipulating participants' cultural background, we manipulated the properties of the melodies and instrument timbre. Future research could include listeners highly familiar with Balinese gamelan. The musical training of listeners from Balinese gamelan and the Western tonal tradition could be crossed with the present musical material and timbre variables, especially as musicians and non-musicians may differ in pitch perception and in their response to inharmonic complexes (McLachlan et al., 2013). A priming paradigm could also be used to gather accuracy and reaction time data, building on the perceptual ratings provided here, aiming to provide further information about musical expectations and their influence on processing speed.

### Conflict of interest statement

The authors declare that the research was conducted in the absence of any commercial or financial relationships that could be construed as a potential conflict of interest.

## Footnotes

^1^http://anasynth.ircam.fr/home/english/software/audiosculpt

## Appendix 1

Lowest frequency partial (Hz) of the original and sister instrument tones used in the experiment. L refers to the note in the lower octave range played on the same instrument.

**Table:**

**Note**	**Original**	**Sister**	**Difference**
1	599.2	607.3	8.1
2	634.1	642.2	8.1
3	694.6	702.6	8.0
4	824.5	833.0	8.5
5	892.1	900.5	8.4
6	947.1	955.2	8.1
7	1051.4	1059.9	8.5
5L	443.3	451.7	8.4
6L	471.1	478.6	7.5

## Appendix 2

Cipher or number notation for musical stimuli composed from the Selisir mode of the pelog system. Cipher notation assigns numbers to the metal keys. Note 1 = i (ding); Note 2 = o (dong), Note 3 = e (deng), Note 5 = u (dung), and Note 6 = a (dang). A dot above a number indicates a higher octave and a dot below indicates a lower octave. Note duration is depicted spatially and, in most instances below, one cell refers to a quaver or 1/8^th^ note. “In Scale” refers to the note from within the scale that replaced the final gong tone in the in-scale trials and “Out” refers to the tone that replaced the final gong tone selected from outside of the scale (Notes 4 or 7) in the completion judgment trials. “Partial” and “Total” refer to few or multiple replacements of the gong tone with an out of scale tone (Note 4) in cohesion judgment trials. A brief description of musical characteristics of five of the melodies is also provided.

### Ding 1

Ding 1 is composed around a recurring four note motif that descends and rises stepwise. The first two notes of the motif are then repeated but in half time. The third and final occurrence of the four-note motif is played a step higher.

The unit of time in Ding 1, as depicted here and based on the shortest note, is that of a quaver, i.e., the opening rhythm of Ding 1 is:

### Ding 2

The unit of time in Ding 2 based on the shortest note is a quaver, i.e., the opening rhythm of Ding 2 is:

### Dong 1

Dong 1 uses a 3-note motif related to create 8-note phrases—returning to itself—the motives overlap at the end/beginning of each 8-note phrase.

Dong 1 is isochronous and the unit of time shown here is a crotchet, e.g.,

### Dong 2

The minimal unit of time in Dong 2 is a quaver, i.e., the opening rhythm of Dong 2 is:

### Deng 1

Deng 1 contains repeated but shifted motives—the rising series of motives toward the end become the opening motif. There are 4 phrases, not all equal lengths.

The time unit in Deng 1 is shown here as a semiquaver (viz. the shortest duration). The opening rhythm is:

### Deng 2

The Deng 2 time unit shown here is a quaver. The opening rhythm is:

### Dung 1

Dung 1 has a syncopated, flowing melody with repeated motives. The third phrase is longer and ends with a series of 3 quaver beat motives throwing the rhythm off balance ready for the return of the gong tone.

The shortest note and the unit of time depicted below is a quaver. The opening rhythm of Dung 1 is:

### Dung 2

The unit of time based on the shortest note in Dung 2 is a quaver. The opening rhythm is:

### Dang 1

Dang 1 has a typical repetitive nature plus a linking phrase (the last 8 notes) to lead back to the gong tone.

Unit length in Dang 1 as depicted here is similar to a quaver, i.e., the opening rhythm of Dang 1 is:

### Dang 2

Unit length in Dang 2 as depicted here is similar to a quaver, i.e., the opening rhythm of Dang 2 is:
